# Intake of whole grain foods and risk of coronary heart disease in US men and women

**DOI:** 10.1186/s12916-022-02396-z

**Published:** 2022-06-10

**Authors:** Yang Hu, Walter C. Willett, Jo Ann E. Manson, Bernard Rosner, Frank B. Hu, Qi Sun

**Affiliations:** 1 Department of Nutrition, Harvard T.H. Chan School of Public Health, 665 Huntington Avenue, Boston, MA 02115 USA; 2 Department of Epidemiology, Harvard T.H. Chan School of Public Health, Boston, MA USA; 3 Channing Division of Network Medicine, Department of Medicine Research, Brigham and Women’s Hospital and Harvard Medical School, Boston, MA USA; 4 Division of Preventive Medicine, Department of Medicine Research, Brigham and Women’s Hospital and Harvard Medical School, Boston, MA USA; 5 Department of Biostatistics, Harvard T.H. Chan School of Public Health, 665 Huntington Avenue, Boston, MA 02115 USA; 6 Joslin Diabetes Center, Boston, MA 02115 USA

**Keywords:** Whole grain food, Coronary heart disease, Prospective cohort study

## Abstract

**Background:**

Epidemiological studies have demonstrated a favorable association of whole grain intake with coronary heart disease (CHD) risk, although whether such an inverse association holds true for individual whole grain foods that have various nutritional profiles has not been examined.

**Methods:**

We followed 74,244 women from Nurses’ Health Study since 1986, 91,430 women from Nurses’ Health Study II since 1991, and 39,455 men from the Health Professionals Follow-Up Study since 1984, who did not have a history of cardiovascular disease or cancer at baseline. Intake of seven individual whole grain foods was repeatedly assessed using a validated semi-quantitative food frequency questionnaire every 2–4 years since baseline. CHD diagnoses were ascertained through review of medical records or death certificates.

**Results:**

We documented 9461 CHD cases during an average of 25.8 years’ follow-up. In the multivariable-adjusted model, the pooled hazard ratio (HR) (95% CI) of CHD risk corresponding to each one serving/day consumption of total whole grains was 0.93 (0.90–0.95; *p* trend <0.0001). Higher consumption of most individual whole grain foods was associated with significantly lower risk of CHD. Comparing participants consuming ≥1 serving/day with those consuming < 1 serving/month, the multivariable-adjusted pooled HRs (95% CIs) of CHD were 0.83 (0.78–0.89) for whole grain cold breakfast cereal, 0.92 (0.86–0.99) for dark bread, and 1.08 (0.96–1.22) for popcorn. For other whole grain foods with lower overall intake levels, comparing intake level of ≥2 servings/week with < 1 serving/month, the pooled hazard ratios (95% CIs) were 0.79 (0.74–0.84) for oatmeal, 0.79 (0.71–0.87) for brown rice, 0.84 (0.78–0.90) for added bran, and 0.87 (0.77–0.99) for wheat germ. Cubic spline regression suggested non-linear associations for certain whole grain foods: the risk reduction plateaued approximately over 2 servings/day for total whole grains, 0.5 serving/day for both cold breakfast cereal and dark bread, 0.5 serving/week for oatmeal, 1 serving/week for brown rice, and 2 serving/week for added bran (*p* for non-linearity <0.01 for all associations).

**Conclusions:**

These data suggest that higher consumption of total whole grains, as well as individual whole grain foods except popcorn, were significantly associated with lower CHD risk. The inverse associations may plateau at various intake levels for total whole grain and individual whole grain foods. This study provides further evidence in support of increasing whole grain intake for the prevention of CHD in US populations.

**Supplementary Information:**

The online version contains supplementary material available at 10.1186/s12916-022-02396-z.

## Background

Coronary heart disease (CHD) remains one of the leading causes of deaths in the USA [1]. CHD is largely preventable through adopting a healthy lifestyle and diet [2]. Of many modifiable dietary factors, whole grains have been extensively examined in relation to risk of CHD. Most epidemiological studies derived total whole grain intake by summing up the whole-grain contents from all food sources [3], and the majority of prospective cohort studies have consistently documented substantial health benefits of overall whole grain consumption on the prevention of CHD [4–8]. However, few studies have specifically examined the relationship between individual whole grain foods and risk of CHD [7–11]. Given various biochemical compositions of grain species, as well as exogenous ingredients introduced during food preparation [12], it is likely that different whole grain foods may exert differential effects on cardiovascular health [13–15]. Recent meta-analyses of epidemiological studies have consistently suggested cardio-protective effects of total wholes as well as several whole grain foods including whole grain cold breakfast cereal, whole grain bread, and added bran, while the associations and dose-response relationship with other commonly consumed whole grain foods, such as brown rice, oatmeal, and popcorn, remain largely unknown [16–18].

To fill the knowledge gap, the current study systematically evaluated the associations between intake of several commonly consumed whole grain foods including whole grain cold breakfast cereal, oatmeal, dark bread, brown rice, popcorn, wheat germ, and added bran and the risk of CHD in three large prospective cohorts of health professionals with diet and disease status repeatedly assessed over three decades of follow-up.

## Methods

### Study population

The Nurses’ Health Study (NHS) was initiated in 1976, when 121,700 female registered nurses aged 30–55 years answered a mailed questionnaire on their medical history and lifestyle characteristics. A parallel cohort study of younger women, the Nurses’ Health Study II (NHSII), was established in 1989 and included 116,340 eligible female nurses aged 25–42 years. A questionnaire similar to that used in NHS was administered at baseline to assess medical history and lifestyle factors. In 1986, the Health Professionals Follow-up Study (HPFS) was started and recruited 51,529 US male health professionals aged 40–75 years. The HPFS participants completed a baseline questionnaire that was similar to that used in the NHS and NHSII. In all three cohorts, participants were sent questionnaires biennially to update their diet and lifestyle information and identify newly diagnosed CHD and other diseases. The cumulative response rates in three cohorts exceeded 90% [19, 20].

The study baseline was set to be 1984 for NHS, 1991 for NHSII, and 1986 for HPFS, when a comprehensive semi-quantitative food frequency questionnaire (sFFQ) was administered to assess diet. The exclusion criteria included the presence of cardiovascular disease or cancer at baseline (*n*=5942 in HPFS, 3869 in NHS, 1104 in NHSII), not returning the sFFQ or had unusual total energy intake (<500 or >3500 kcal/day for women and <800 or >4200 kcal/day for men) (*n*=1595 in HPFS, 15,753 in NHS, 13,001 in NHSII), or completing baseline questionnaire only (*n*=694 in HPFS, 523 in NHS, 624 in NHSII). The final study population consisted of 74,244 participants in NHS, 91,430 in NHSII, and 39,455 in HPFS.

The study protocol was approved by the Human Research Committee of Brigham and Women’s Hospital and the Harvard T.H. Chan School of Public Health. Completion and return of study questionnaires implied informed consent of the participants.

### Dietary assessment

In all three cohorts, diet was assessed using the validated sFFQ at baseline and updated every 2–4 years during the follow-up until 2014 in NHS, 2015 in NHSII, and 2016 in HPFS. For each food item listed in the sFFQ, the participants were asked their average consumption frequency of a pre-specified portion size during the previous year. There are nine possible responses for consumption frequencies ranging from never or <1 time/month to ≥6 times/day. In the current analysis, we focused on the consumption of seven commonly consumed whole grain foods/components, including cold breakfast cereals (serving size, 1 cup), dark bread (serving size, 1 slice), popcorn (serving size, 1 cup), oatmeal (serving size, 1 cup), bran added to food (serving size, 1 teaspoon), wheat germ (serving size, 1 teaspoon), and brown rice (serving size, 1 cup). Based on the brand names provided by the participants, we further classified cold breakfast cereal into whole grain-based breakfast cereal and refined grain-based breakfast cereal, depending on the relative contents of whole grain ingredients in the product (containing ≥25% whole grains or bran by weight classified as whole grain cold breakfast cereal). Since 2002 in the NHS and HPFS, and 2003 in the NHSII, an additional question regarding the types of popcorn was added to the sFFQ in which participants were asked whether they consumed regular or light/fat free popcorn. We estimated the total whole grain consumption by first transforming the reported serving size into grams and then summed up the weight of whole grain ingredients according to corresponding whole grain contributions in all grain-containing food [21]. The seven individual whole grain foods contributed on average 86% of total whole grain in NHS, 78% in NHSII, and 84% in HPFS during the follow-up. Validation studies showed the validity of whole grain foods assessments. For example, the FFQ assessments were significantly correlated with those assessed using multiple-day diet records; the correlation coefficients were 0.58 for dark bread and 0.73 for cold breakfast cereal [22].

### Demographic and lifestyle factors assessment

In all three cohorts, a similar follow-up questionnaire was mailed to participants to assess and update information regarding smoking status, vitamin supplements use, alcohol consumption, menopausal status (women only), and years of postmenopausal hormone use (women only), physician-diagnosed hypertension and hypercholesterinemia, and other time-varying variables. Height was reported at baseline, and body weight was updated biennially. Body mass index (BMI) was calculated as weight in kilograms divided by the square of height in meters (kg/m^2^). Recreational physical activity was measured with a validated questionnaire asking about the average time spent on 10 common activities. Based on this information, we calculated weekly energy expenditure in metabolic equivalent (METs) hours weighting each activity by its intensity level [23]. Multiple validation studies demonstrated adequate validity of these self-reported variables [24–28]. We used the alternative healthy eating index (AHEI) [29], after removing the whole grain component, to represent overall diet quality.

### Assessment of coronary heart disease

Total CHD including nonfatal myocardial infarction (MI) and fatal CHD was the primary disease outcome for the current analysis. In all three cohorts, permission was sought to access medical records of participants who reported having a nonfatal MI on a follow-up questionnaire. Study physicians who were blinded to exposure status reviewed the medical records and confirmed a reported MI according to the WHO criteria, which require the presence of symptoms, and either typical electrocardiographic changes or elevated cardiac enzyme levels [30, 31]. Deaths were identified through reports from the next of kin, the postal authorities, or by searching the National Death Index (NDI) [32]. Fatal CHD was confirmed by a review of hospital records or autopsy reports if CHD was listed as the underlying cause of death and if evidence of previous CHD was available from medical records [33]. Sudden deaths without cardiac causes were not considered as fatal CHD in the current analysis.

### Statistical analysis

Cumulative averages of total whole grains and individual whole grain foods were calculated to represent long-term intake using the formula ${Y}_n=\frac{\sum_i^n{Y}_i}{n}$, where *Y*_*n*_ denotes the intake level at nth follow-up cycle [34]. For each participant, we counted their person-time from the return date of the baseline FFQ to the CHD diagnosis date, death date, date of last return of a valid follow-up questionnaire, or the end of follow-up (2014 for NHS, 2016 for HPFS, and 2017 for NHSII), whichever occurred first. To alleviate the potential reverse causality that participants with existing diseases might change their usual diet intake that is etiologically relevant, we stopped updating dietary information once the participants developed diabetes, stroke, coronary artery bypass graft, hypertension, hypercholesterinemia, or cancer during follow-up. The proportion of missing values of covariates ranged from 4.6% for smoking status to 5.1% for BMI. We replaced missing values with valid values in the preceding questionnaire for one follow-up cycle and then created missing indicators to handle subsequent missing values.

An age- (months) and calendar time-stratified multivariable-adjusted Cox proportional hazards model was used to estimate the hazard ratios (HRs) and 95% confidence intervals (CIs) for the association between total whole grains as well as individual whole grain foods and risk of CHD. The risk set was defined by both age and calendar year which minimized the potential confounding effects by age and time trend and accommodated the time-varying modeling at the same time. The proportional hazards assumption was evaluated by including product terms between each categorical whole grain variable and the duration of follow-up calculated as months from baseline to CHD diagnosis date, death, or the end of follow-up, whichever occurred first. The proportional hazards assumption was evaluated by including product terms between each categorical whole grain variable and the duration of follow-up calculated as months from baseline to CHD diagnosis date, death, or the end of follow-up, whichever occurred first. The likelihood ratio tests were used to compare models with and without interaction terms and none of the *p* values were statistically significant in pooled results, suggesting no violations of the proportional hazard assumption. Total whole grain intake was categorized into fifths using quintiles according to cohort-specific distributions in each follow-up cycle. We used three pre-specified categories (< 1 serving/month, 1 serving/month to 1 serving/week, and ≥ 2 servings/week) for oatmeal, brown rice, added bran, and wheat germ which had modest-to-low intake, whereas four pre-specified categories (< 1 serving/month, 1 serving/month to 1 serving/week, 1 serving/week to 4–6 servings/week, and ≥ 1 serving/day) were used for whole grain cold breakfast cereal, dark bread, and popcorn, for which the consumption level were on average higher. Covariates included in the models were ethnicity (white, African American, Asian, others), time-varying BMI (<21.0, 21.0–22.9, 23.0–24.9, 25.0–26.9, 27.0–29.9, 30.0–32.9, 33.0–34.9, or ≥35.0 kg/m^2^), smoking status (never smoked, past smoker, currently smoke 1–14 cigarettes per day, 15–24 cigarettes per day, or ≥25 cigarettes per day), alcohol intake (0, 0.1–4.9, 5.0–9.9, 10.0–14.9, 15.0–29.9, and ≥30.0 g/day), multivitamin use (yes, no), physical activity (quintiles), modified AHEI (quintiles), total energy (quintiles), family history of myocardial infarction (yes, no), baseline diabetes (yes, no), postmenopausal hormone use (women only; never, former, or current hormone use, or missing), and oral contraceptive use (yes, no; women only). The continuous variable of total whole grain consumption and each individual whole grain food was used to calculate *p* value for trend and HR (95% CI) of CHD per one serving/day of intake. We also evaluated the substitution effects of replacing 10 g of total refined grain with the same amount of total whole grain on CHD risk by mutually adjusting two variables in the same model. Differences in their *β* coefficients were used to estimate the HRs for the substitution association, and their variances and covariance matrix were used to derive the 95% CI [34].

We evaluated the heterogeneous associations among individual whole grain foods with CHD risk using a likelihood ratio test comparing two models after adjusting for other covariates: one with total whole grains only and the other with total whole grains plus seven individual whole grain foods in categorical terms. Moreover, in the subgroup analysis, we explored the effect modification by several lifestyle factors, including BMI, physical activity, smoking status, and family history of myocardial infarction. *P* values for interactions were calculated from likelihood ratio tests comparing two nested models: the full models with product terms between quintile of total whole grain and categorical lifestyle factors and the reduced models without the product terms. In a secondary analysis, we analyzed the associations for regular or fat-free/light popcorn consumption with CHD risk. Data from each cohort were analyzed separately, and results were pooled using a fixed-effects model. Finally, to delineate the dose-response relationship between whole grain and CHD risk, we combined data from three cohorts and fitted cubic spline regressions with the same covariate adjustment in the primary analysis (except for women only variables) for total whole grain and individual whole grain foods. The whole grain intake levels were truncated at 99.5th percentile to avoid the influence of extreme values. Total whole grain intake was converted to servings by dividing a factor of 16 according to the dry weight estimation of serving size [35, 36]. Corresponding to 5th, 35th, 65th, and 95th percentile of total whole grain intake, four knots at 0.17, 0.76, 1.4, and 2.9 servings/day were used for the cubic splines. We used likelihood ratio tests with 2 degree of freedom (knots number −2) to calculate *p* value for non-linearity by comparing models with linear term only and models with both linear and spline terms.

Several sensitivity analyses were performed. First, because the strategy of stopping updating dietary information upon occurrence of chronic conditions may introduce differential errors in dietary assessments between diseased participants and other participants, we conducted a sensitivity analysis by resuming to update diet 8 years after the incidence of these intermediate outcomes. For example, for participants who developed stroke in 1990, we stopped updating diet in 1994 and 1998 and then continued to update diet after 1998. Second, we used the baseline intake or the simple-updated time-varying intake instead of the cumulative averaged value to repeat the analyses. Finally, we conducted a 4-year latency analysis to address the potential reverse causation bias. All statistical tests were 2-sided with significant level of 0.05 and performed using SAS 9.3 (SAS Institute, Cary, NC).

## Results

Table 1 presents participants’ baseline characteristics according to the total whole grain intake in the three cohorts. In all three cohorts, higher total whole grain intake correlated with a constellation of healthy lifestyle and dietary factors, including a lower BMI, higher physical activity level, lower prevalence of smoking, higher prevalence of multivitamin use, and better diet quality. Moreover, at baseline frequent whole grain consumers tended to have a lower prevalence of hypertension and a higher prevalence of hypercholesterolemia except for participants in the NHSII for whom whole grain intake was associated with a lower prevalence of both conditions. Although all individual whole grain foods intakes were modestly correlated with total whole grain intake, the Pearson correlation coefficients among individual whole grain foods were mostly weak except a moderate correlation (*r*=0.29) found between added bran and wheat germ (Additional file 1: Table S1).

**Table 1:** Age-standardized characteristics of study participants in Nurses’ Health Study (1984–2014), Nurses’ Health Study II (1991–2017), and Health Professionals Follow-up Study (1986–2016)

	Total whole grain consumption				
	Q1	Q2	Q3	Q4	Q5
**Nurses’ Health Study**					
No of participants	15,033	14,567	14,858	14,925	14,861
Total whole grain, servings/day	0.1 (0.1, 0.2)	0.4 (0.3, 0.4)	0.7 (0.6, 0.7)	1 (0.9, 1.2)	1.9 (1.6, 2.4)
Total whole grain, g/day	2.3 (1.3, 3.3)	6.1 (5.2, 7)	10.4 (9.2, 11.7)	16.7 (14.8, 18.8)	29.9 (24.9, 38.8)
Age (years)	49.8 (6.9)	49.9 (7.1)	50.3 (7.1)	51.2 (7.2)	52.5 (7.2)
Baseline body mass index (kg/m^2^)	25.2 (5)	25.3 (5)	25.2 (4.7)	24.9 (4.6)	24.4 (4.3)
Race					
White, %	96.8	97.8	98.1	98.1	98.2
African American, %	0.3	0.2	0.2	0.2	0.3
Asian, %	1.2	0.7	0.6	0.5	0.4
Others, %	1.7	1.2	1.1	1.2	1.1
Physical activity (MET-h/week)^a^	5.1 (1.9, 15.2)	6.8 (2.4, 16.5)	7.7 (2.9, 18.5)	8.4 (3.1, 20.2)	9.9 (3.4, 21.5)
Hypertension, %	9.2	8.4	7.7	7.2	6.8
High cholesterol, %	3.0	3.0	3.2	3.6	4.1
Baseline diabetes, %	0.9	0.8	0.8	0.9	1.0
Family history of MI, %	19.9	19.9	19.6	18.4	19.4
Current smokers, %	36.2	27.6	23.3	19.1	14.5
Multivitamin use, %	30.3	34.1	36.8	39.8	44.3
Oral contraceptive use, %	46.7	48.7	49.6	49.7	49.9
Hormone use					
premenopausal, %	52.1	52.5	53.2	52.6	52.4
postmenopausal-never, %	27.4	25.8	24.6	23.7	22.8
postmenopausal-current, %	9.1	10.9	11.1	12.4	13.7
postmenopausal-past, %	11.4	10.8	11.0	11.4	11.2
Alcohol consumption (g/day)^a^	2.8 (0, 12.5)	2.7 (0, 10.8)	2.6 (0, 9.5)	2 (0, 7.8)	1.8 (0, 5.8)
Total energy intake (kcal/day)	1710.5 (553)	1790.1 (541.4)	1781.9 (540.7)	1775.5 (523)	1651.4 (475.5)
Modified alternative healthy eating index	43.4 (9.9)	44.8 (9.7)	46.3 (9.8)	47.5 (10)	50 (10.6)
**Nurses’ Health Study II**					
No of participants	18,384	18,203	18,280	18,345	18,218
Total whole grain, servings/day	0.3 (0.2, 0.4)	0.7 (0.6, 0.8)	1.1 (1, 1.2)	1.6 (1.4, 1.7)	2.5 (2.2, 3.1)
Total whole grain, g/day	4.9 (3.1, 6.5)	10.9 (9.5, 12.3)	17 (15.4, 18.7)	24.8 (22.5, 27.5)	40.1 (34.6, 49.3)
Age (years)	36.6 (4.7)	36.5 (4.7)	36.5 (4.7)	36.5 (4.6)	36.9 (4.6)
Baseline body mass index (kg/m^2^)	25.1 (5.9)	25 (5.6)	24.7 (5.3)	24.4 (5)	23.9 (4.6)
Race					
White, %	94.3	96.2	97.1	97.5	97.3
African American, %	0.5	0.6	0.4	0.4	0.6
Asian, %	3.1	1.4	1.1	0.9	1.0
Others, %	2.1	1.8	1.3	1.1	1.1
Physical activity (MET-h/week)^a^	9.4 (3.4, 22)	11.3 (4.5, 24.6)	12.6 (5.2, 25.9)	13.9 (5.9, 28.4)	16.5 (7.3, 33)
Hypertension, %	4.4	3.8	3.2	3.0	2.6
High cholesterol, %	10.1	9.8	9.4	9.1	9.0
Baseline diabetes, %	0.7	0.6	0.6	0.5	0.8
Family history of MI, %	49.6	49.7	48.8	48.4	48.2
Current smokers, %	19.6	14.5	11.4	8.6	7.2
Multivitamin use, %	36.1	40.7	44.1	47.7	50.7
Oral contraceptive use, %	82.5	84.6	84.7	84.4	83.3
Hormone use					
premenopausal, %	97.1	96.8	97.1	97.0	96.7
postmenopausal-never, %	0.2	0.2	0.2	0.2	0.2
postmenopausal-current, %	2.4	2.8	2.4	2.6	2.8
postmenopausal-past, %	0.3	0.3	0.2	0.2	0.3
Alcohol consumption (g/day)^a^	0.9 (0, 3)	0.9 (0, 3.7)	0.9 (0, 3.7)	0.9 (0, 3.5)	0.9 (0, 3)
Total energy intake (kcal/day)	1771.7 (578)	1821.1 (561.2)	1823.9 (550.8)	1820 (526.8)	1709.8 (509.6)
Modified alternative healthy eating index	44.7 (10)	44.8 (9.9)	45.1 (10)	45.3 (9.9)	45.9 (10.2)
**Health Professionals Follow-up Study**					
No of participants	7948	7892	7818	7892	7905
Total whole grain, servings/day	0.2 (0.1, 0.3)	0.6 (0.5, 0.7)	1.1 (0.9, 1.2)	1.7 (1.5, 1.9)	2.8 (2.4, 3.6)
Total whole grain, g/day	3.4 (1.6, 5.1)	9.9 (8.3, 11.6)	16.9 (15, 19)	26.6 (23.7, 29.9)	45.5 (38.8, 57.6)
Age (years)	52.5 (9.2)	52 (9.3)	52.4 (9.3)	53 (9.5)	53.8 (9.5)
Baseline body mass index (kg/m^2^)	25.8 (3.3)	25.8 (3.3)	25.6 (3.3)	25.4 (3.3)	24.8 (3)
Race					
White, %	92.9	94.9	95.9	96.8	95.4
African American, %	2.3	2.3	2.1	1.8	2.4
Asian, %	3.6	1.4	1.0	0.9	1.3
Others, %	1.1	1.4	0.9	0.5	0.9
Physical activity (MET-h/week)^a^	8.5 (2.5, 23.1)	10.9 (3.5, 26.3)	12.3 (4.4, 27.7)	13.9 (4.8, 29.4)	16.7 (6.3, 35.1)
Hypertension, %	22.2	20.8	18.3	18.0	18.5
High cholesterol, %	9.2	9.5	10.0	10.3	12.8
Baseline diabetes, %	2.1	2.1	2.3	2.4	2.9
Family history of MI, %	12.4	12.3	11.9	11.5	11.9
Current smokers, %	17.7	12.5	8.6	6.2	4.5
Multivitamin use, %	34.7	37.8	41.2	45.1	50.3
Alcohol consumption (g/day)^a^	6.9 (1, 18.8)	6.6 (1.1, 16.5)	6.4 (1, 16.1)	5.5 (0.9, 13.7)	3.9 (0, 12)
Total energy intake (Kcal/day)	1986.9 (646.9)	2036 (642.9)	2062.9 (637.6)	2021 (595.9)	1880.5 (556.3)
Modified alternative healthy eating index	45.9 (10.6)	48.5 (10.3)	49.9 (10.4)	51.5 (10.5)	54.7 (10.3)

Values are means (SD) or percentages and are standardized to the age distribution of the study population

^a^Value is median (interquartile range)

During an average follow-up duration of 25.8 years, a total of 9461 CHD cases were documented, of which 5878 were nonfatal MI and 3583 cases were fatal CHD. In all three cohorts, higher total whole grain consumption (serving size, 16 g/serving) was associated with a significantly lower risk of CHD (Table 2). Comparing extreme quintiles, the multivariable-adjusted pooled HR was 0.77 (95% CI 0.72–0.82, *p* trend <0.0001). Replacing each 10 g of total refined grain with 10 g of total whole grain was associated with 3% (95% CI: 2–5%) lower risk of CHD. The associations between individual whole grain foods and CHD risk are presented in Table 3. In the pooled results, comparing participants consuming ≥1 serving/day with those consuming < 1 serving/month, the multivariable-adjusted pooled HRs (95% CIs) of CHD were 0.83 (0.78–0.89) for whole grain cold breakfast cereal, 0.92 (0.86–0.99) for dark bread, and 1.08 (0.96–1.22) for popcorn. For other whole grain foods with lower overall intake levels, comparing intake level of ≥2 servings/week with < 1 serving/month, the pooled HRs (95% CIs) were 0.79 (0.74–0.84) for oatmeal, 0.79 (0.71–0.87) for brown rice, 0.84 (0.78–0.90) for added bran, and 0.87 (0.77–0.99) for wheat germ. Results by cohorts are shown in Additional file 1: Table S2.

**Table 2:** Pooled HRs (95% confidence intervals) of coronary heart disease for total whole grains consumption in Nurses’ Health Study (1984–2014), Nurses’ Health Study II (1991–2017), and Health Professionals Follow-up Study (1986–2016)

	Total whole grain consumptions					*P* trend	Per one daily serving
	Q1	Q2	Q3	Q4	Q5		
**NHS**							
Median, g/day	3.2	7.8	12.5	18.9	34.4		
Cases/person years	1010/397,202	790/396,834	722/398,587	669/399,124	710/398,560		
Age-adjusted model	1.00	0.78 (0.71, 0.86)	0.69 (0.63, 0.76)	0.61 (0.55, 0.67)	0.58 (0.52, 0.64)	<.0001	0.81 (0.77, 0.85)
Multivariable-adjusted model^a^	1.00	0.88 (0.80, 0.96)	0.84 (0.76, 0.92)	0.79 (0.71, 0.87)	0.79 (0.71, 0.88)	0.002	0.93 (0.89, 0.97)
**NHSII**							
Median, g/day	6.7	13.7	20.1	27.7	41.1		
Cases/person years	263/465,042	196/465,604	142/466,968	107/467,520	109/466,930		
Age-adjusted model	1.00	0.79 (0.65, 0.95)	0.58 (0.47, 0.71)	0.43 (0.34, 0.54)	0.42 (0.34, 0.53)	<.0001	0.66 (0.60, 0.73)
Multivariable-adjusted model^a^	1.00	0.93 (0.77, 1.12)	0.77 (0.63, 0.95)	0.62 (0.49, 0.79)	0.63 (0.50, 0.80)	<.0001	0.80 (0.73, 0.87)
**HPFS**							
Median, g/day	4.6	12.0	19.7	29.3	46.8		
Cases/person years	1182/193,927	978/194,734	888/195,423	845/195,433	850/195,576		
Age-adjusted model	1.00	0.84 (0.77, 0.91)	0.75 (0.69, 0.82)	0.69 (0.63, 0.76)	0.66 (0.60, 0.72)	<.0001	0.89 (0.87, 0.92)
Multivariable adjusted model^a^	1.00	0.89 (0.81, 0.97)	0.83 (0.76, 0.90)	0.80 (0.73, 0.88)	0.77 (0.71, 0.85)	<.0001	0.94 (0.91, 0.96)
**Pool results**^b^							
Cases/person years	2455/1,056,171	1964/1,057,172	1752/1,060,978	1621/1,062,077	1669/1,061,066		
Age-adjusted model	1.00	0.81 (0.76, 0.86)	0.71 (0.67, 0.75)	0.63 (0.60, 0.68)	0.60 (0.57, 0.64)	<.0001	0.86 (0.84, 0.87)
Multivariable-adjusted model	1.00	0.89 (0.84, 0.94)	0.83 (0.78, 0.88)	0.78 (0.73, 0.83)	0.77 (0.72, 0.82)	<.0001	0.93 (0.90, 0.95)

^a^Adjusted for age (years), ethnicity (white, African American, Asian, others), updated body mass index (calculated as weight in kilograms divided by height in meters squared) (<21.0, 21.0–22.9, 23.0–24.9, 25.0–26.9, 27.0–29.9, 30.0–32.9, 33.0–34.9, or ≥35.0 kg/m^2^), smoking status (never smoked, past smoker, currently smoke 1–14 cigarettes per day, 15–24 cigarettes per day, or ≥25 cigarettes per day), alcohol intake (0, 0.1–4.9, 5.0–9.9, 10.0–14.9, 15.0–29.9, and ≥30.0 g/day), baseline diabetes (yes, no), multivitamin use (yes, no), physical activity (quintiles), modified alternative healthy eating index (quintiles, whole grain component was excluded), total energy (quintiles), and family history of MI (yes, no). For women, postmenopausal hormone use (never, former, or current hormone use, or missing) and oral contraceptive use (yes, no) were further adjusted

^b^Study estimates from three cohorts were pooled using a fixed effects model

**Table 3:** Pooled hazard ratios (95% confidence intervals) of coronary heart disease for individual whole grain food consumption in Nurses’ Health Study (1984–2014), Nurses’ Health Study II (1991–2017), and Health Professionals Follow-up Study (1986–2016)

	Consumptions				*P* trend	Per one daily serving
	< 1 serving/month	1 serving/month to 1 serving/week	1 serving/week to 4–6 servings/week	≥1 servings/day		
Whole grain cold breakfast cereal						
Cases/person-time	3819/1,659,126	1805/1,069,850	2485/1,875,639	1352/692,849		
Multivariable adjusted model^a^	1.00	0.91 (0.86, 0.96)	0.75 (0.71, 0.79)	0.83 (0.78, 0.89)	<.0001	0.81 (0.77, 0.87)
Dark bread						
Cases/person-time	1192/585,183	2067/1,070,944	2826/1,946,391	3376/1,694,945		
Multivariable adjusted model^a^	1.00	1.04 (0.97, 1.12)	0.86 (0.80, 0.92)	0.92 (0.86, 0.99)	0.41	0.99 (0.96, 1.01)
Popcorn						
Cases/person-time	3522/1,274,468	4218/2,662,637	1381/1,151,269	340/209,090		
Multivariable adjusted model^a^	1.00	0.98 (0.94, 1.03)	0.92 (0.86, 0.98)	1.08 (0.96, 1.22)	0.73	1.01 (0.94, 1.09)
	< 1 serving/month	1 serving/month to 1 serving/week	≥2 servings/week			
Oatmeal						
Cases/person-time	5243/2,601,706	3063/1,930,893	1155/764,866			
Multivariable adjusted model^a^	1.00	0.95 (0.90, 0.99)	0.79 (0.74, 0.84)		0.007	0.84 (0.75, 0.95)
Brown rice						
Cases/person-time	6002/3,021,014	3039/1,913,498	420/362,950			
Multivariable adjusted model^a^	1.00	0.92 (0.88, 0.97)	0.79 (0.71, 0.87)		0.02	0.76 (0.61, 0.95)
Added bran						
Cases/person-time	7456/4,184,117	1086/644,738	919/468,610			
Multivariable adjusted model^a^	1.00	0.92 (0.87, 0.99)	0.84 (0.78, 0.90)		0.007	0.91 (0.85, 0.98)
Wheat germ						
Cases/person-time	8645/4,813,315	540/344,426	276/139,724			
Multivariable adjusted model^a^	1.00	0.95 (0.87, 1.04)	0.87 (0.77, 0.99)		0.06	0.86 (0.74, 1.01)

^a^Adjusted for age (years), ethnicity (white, African American, Asian, others), body mass index (calculated as weight in kilograms divided by height in meters squared) (<21.0, 21.0–22.9, 23.0–24.9, 25.0–26.9, 27.0–29.9, 30.0–32.9, 33.0–34.9, or **≥**35.0 kg/m^2^), smoking status (never smoked, past smoker, currently smoke 1–14 cigarettes per day, 15–24 cigarettes per day, or **≥**25 cigarettes per day), alcohol intake (0, 0.1–4.9, 5.0–9.9, 10.0–14.9, 15.0–29.9, and **≥**30.0 g/day), baseline diabetes (yes, no), multivitamin use (yes, no), physical activity (quintiles), modified alternative healthy eating index (quintiles, whole grain component was excluded), total energy (quintiles), and family history of MI (yes, no). For women, postmenopausal hormone use (never, former, or current hormone use, or missing) and oral contraceptive use were further adjusted

Study estimates from three cohorts were pooled using a fixed effects model

**Fig. 1:**
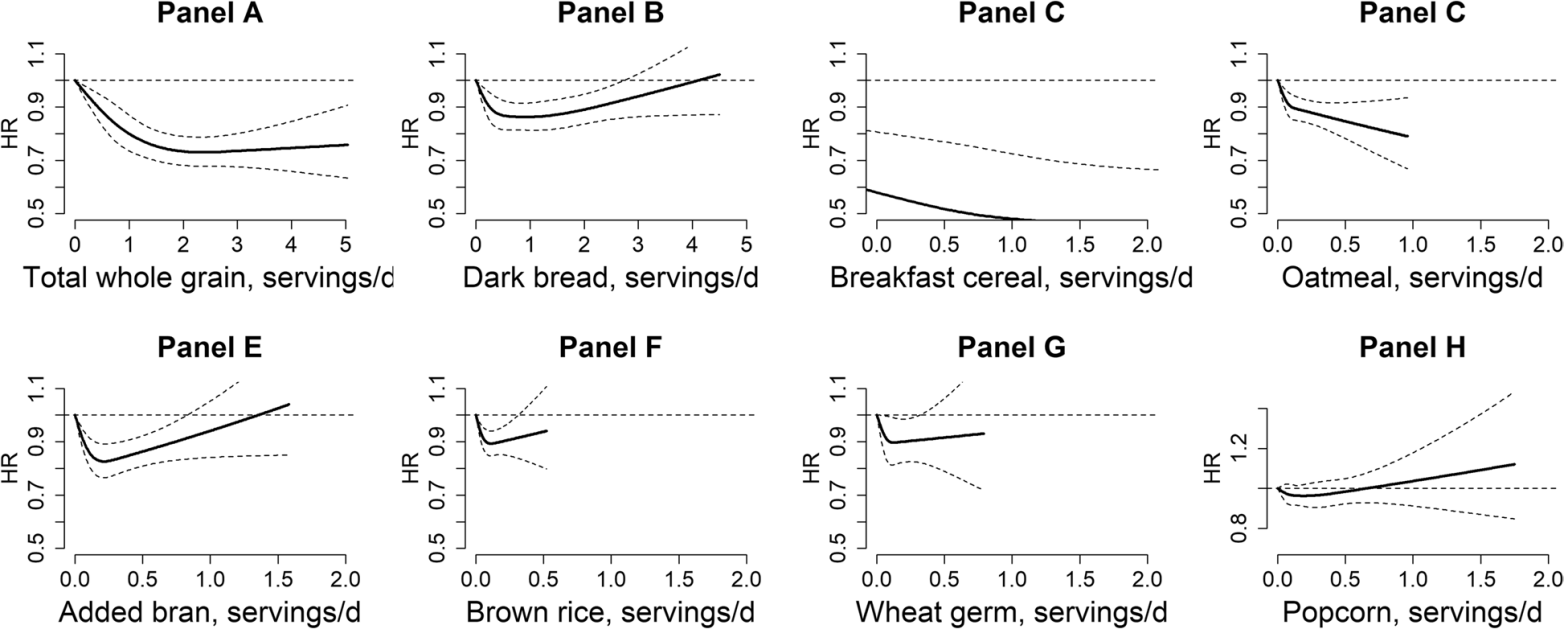
Dose-response relationship for total whole grain and individual whole grain foods in relation to coronary heart disease risk. Data from three cohorts were combined and truncated at 99.5th percentile. Dashed lines were 95% confidence intervals. Models were age- (months) and calendar-time stratified and adjusted for ethnicity (white, African American, Asian, others), updated body mass index (calculated as weight in kilograms divided by height in meters squared) (<21.0, 21.0–22.9, 23.0–24.9, 25.0–26.9, 27.0–29.9, 30.0–32.9, 33.0–34.9, or ≥35.0 kg/m^2^), smoking status (never smoked, past smoker, currently smoke 1–14 cigarettes per day, 15–24 cigarettes per day, or ≥25 cigarettes per day), alcohol intake (0, 0.1–4.9, 5.0–9.9, 10.0–14.9, 15.0–29.9, and ≥30.0 g/day), baseline diabetes (yes, no), multivitamin use (yes, no), physical activity (quintiles), modified alternative healthy eating index (quintiles, whole grain component was excluded), total energy (quintiles), and family history of MI (yes, no). *P* value for non-linearity <0.01 for **A**–**F**, 0.08 for **G**, and 0.30 for **H**

Cubic spline regressions suggested statistically significant non-linear associations for total whole grains (Fig. 1A), dark bread (Fig. 1B), whole grain cold breakfast cereal (Fig. 1C), oatmeal (Fig. 1D), added bran (Fig. 1E), and brown rice (Fig. 1F) in relation to CHD risk, while the test for curvature was not significant for wheat germ (Fig. 1G) or popcorn (Fig. 1H). The CHD risk reduction appeared to plateau approximately over 2 servings/day of total whole grain intake, 0.5 serving/day of dark bread and cold breakfast cereal, 0.5 serving/week for oatmeal, 1 serving/week for brown rice, and 2 servings/week for added bran (*p* for non-linearity <0.01 for all these associations).

The goodness of fit of the fully adjusted model was significantly improved by additionally adjusting for individual whole grain foods (*p*<0.0001 in three cohorts combined) suggesting potential heterogeneity in the associations these foods with CHD risk. The associations were largely similar for nonfatal MI (Additional file 1: Table S3**)** and fatal CHD (Additional file 1: Table S4**)**. In a secondary analysis that examined the associations with regular popcorn and light/fat free popcorn separately, neither type of popcorn was associated with risk of CHD (Additional file 1: Table S5). Stratified analyses did not show differential associations by body weight, physical activity, family history of myocardial infarction, or smoking status (Additional file 1: Figure S1).

In the sensitivity analysis that used baseline intake only or simply-updated intake, the HRs were somewhat attenuated while the estimates were similar in the 4-year latency analysis (Additional file 1: Table S6). Resuming dietary update after 8 years of chronic diseases occurrence produced slightly attenuated but still statistically significant estimates for most whole grain foods (Additional file 1: Table S7).

## Discussion

In three US cohorts of men and women, during over 30 years of follow-up, we found that one daily serving consumption of total whole grains (serving size, 16 g/serving) was associated with a 7% lower risk of CHD, although the risk reduction may plateau over two servings/day of intake. Such inverse associations did not vary by physical activity, smoking status, BMI, or family history of MI. Higher consumption of all individual whole grain foods except popcorn was associated with significant lower risk of CHD while most of these inverse associations appeared to reach a nadir at various intake levels ranging from 0.5 serving/day for dark bread and breakfast cereal to 0.5 serving/week for oatmeal.

The epidemiological evidence consistently supports a favorable role of whole grain intake in CHD prevention. The risk estimates in the current study were largely consistent with two earlier studies involving NHS (1984–1994) and HPFS (1986–2004), respectively [7, 8]. It is noticeable that the inverse associations for total whole grain consumption appeared to be stronger in NHSII which consisted of younger participants than those in NHS and HPFS, suggesting that increasing whole grain intake in early adulthood may be particularly important to cardiovascular health in later life. In the Atherosclerosis Risk in Communities Study, another prospective cohort study of US participants, the hazard ratio (95% CI) of developing CHD was 0.72 (0.53–0.97) comparing participants in the extreme quintiles of total whole grain consumption (average intake level 0.1 serving/day in Q1 vs 3 servings/day in Q5), which was fairly comparable with our pooled study estimates [6, 16]. A meta-analysis of 45 prospective cohort studies estimated that three-serving daily whole grains intake was associated with nearly 20% lower risk of CHD, and there was no evidence of heterogeneity among included studies [17]. Results from our cubic spline regressions confirmed a significant risk reduction up to around 2 servings/day of total whole grain intake; however, we did not see tendency of further risk reduction in higher intake level as opposed to the finding of this meta-analysis. In addition, our study also identified non-linear dose-response relationships for most individual whole grain foods except popcorn. The lowest hazard ratios appeared to be around 0.5 serving/day for cold breakfast cereal and dark bread, 0.5 serving/week for oatmeal, 1 serving/week for brown rice, and 2 servings/week for added bran. Of note, because the consumption level was low for oatmeal, added bran, brown rice, and wheat germ, the risk trajectories at higher consumption levels of these foods remain to be characterized.

In contrast to the abundance of evidence regarding the association between total whole grain intake and health outcomes, there were only a limited number of studies that evaluated associations for individual whole grain foods. The previous investigations were restricted to whole grain breakfast cereal, whole grain-based bread, or added bran, all of which were associated with a lower risk of CHD in prospective studies [7–9]. In the current study, the inverse associations between total whole grain intake and individual whole grain foods and CHD risk were in line with existing evidence linking whole grain intake with cardiovascular risk factors. A meta-analysis of 18 randomized clinical trials (RCTs) concluded that whole grain-based diet rich in beta-glucan, including whole wheat, oat-based breakfast cereal, oatmeal, and oat bran-supplemented foods, was effective in lowering both systolic and diastolic blood pressure [37]. Another meta-analysis including 28 RCTs examining oat bran-enriched diets showed that adding ≥3 g oat beta-glucan to the diet were able to reduce LDL and total cholesterol [38]. Moreover, comparing with a control diet that was mainly white wheat bread, whole grain meals, including whole wheat cookies, whole grain rye bread or rye porridge, and whole grain wheat or rye kernels, led to lower postprandial glucose and insulin response based on data from 14 short-term feeding trials [39]. Furthermore, comparing with refined grain-based food, whole grain food consumption has been shown to reduce inflammatory markers such as high-sensitive C-reactive protein, intercellular adhesion molecule-1, and tumor necrosis factor-α in several clinical intervention studies [40, 41]. Further studies are warranted to integrate genomic, human gut microbiome, and metabolomic data to elucidate the biological pathways underlying the favorable associations of total and individual whole grain foods with cardiometabolic health.

In addition to phytochemical composition intrinsic to different whole grain foods, exogenous ingredients introduced during food processing could also modulate the associations. The null association between popcorn intake and CHD risk may at least partially reflect some adverse effects resulting from constituents introduced during cooking. For example, oil-popped popcorn is prepared with butter, partially hydrogenated oil, and salt. Higher sodium intake is consistently associated with increased blood pressure which is a strong risk factor for CHD development [42, 43]. Despite a declining trend of *trans* fat in US food products, a US food market survey in 2011 still found that popcorn products contained an average of 1.5g/serving of *trans* fatty acids [44], which are associated with an elevated risk of cardiovascular disease [45]. Furthermore, recent studies revealed the presence of perfluoroalkyl substances in microwave popcorn packaging [46, 47]. In a recent investigation, we observed a significant correlation between popcorn intake and blood levels of these endocrine disrupting chemicals, which were associated with type 2 diabetes risk in the NHSII cohort [48]. These ingredients or contaminants may exert adverse effects on lipid profiles, blood pressure, and insulin resistance, and attenuate potential beneficial effects of popcorn on cardiovascular health. In the pooled results of the secondary analysis, although we did not find significant associations for both regular and light/fat free popcorn in relation to CHD risk, we did observe a strong positive association for regular popcorn intake in NHSII. Therefore, it remained to be investigated whether higher regular popcorn intake is particularly unfavorable for the cardiometabolic health of younger women.

The major strengths of the current study include the comprehensive, repeated measurements of several common whole grains foods, large sample size with long follow-up time, and high follow-up rates. Our study has several limitations. First, measurement errors may present in the assessments of individual whole grain foods, particularly for those with low consumptions. However, using the cumulative averages of the individual whole grain foods intake helped to minimize random measurement errors and concomitantly provided estimates of long-term intake of these whole grain foods. Moreover, the measurement error of dietary assessment was likely to be non-differential with respect to the outcome ascertainment due to the prospective design, which usually results in an attenuated association towards the null. Second, despite that we adjusted for a number of potential confounders in the statistical models, the possibility of residual confounding by healthy lifestyle and eating cannot be ruled out. Third, we were unable to analyze the association of other whole grains, such as quinoa, rye, and buckwheat for which the consumption level is low in US populations. Fourth, although stopping updating the dietary information upon occurrence of chronic conditions may help alleviate reverse causation due to changes in diet as a result of the diagnosis of the chronic diseases, this approach may also potentially introduce extraneous heterogeneity in dietary assessments. Nonetheless, in the sensitivity analysis where we stopped updating dietary information until 8 years after occurrence of chronic diseases, the associations were not materially changed, suggesting potentially limited impact of this approach on the associations of interest. Finally, the generalizability of our findings may be limited because the participants were health professionals with higher health consciousness and better access to health care resources.

## Conclusions

In conclusion, higher intake of total whole grains, as well as most commonly consumed individual whole grain foods except popcorn, is associated with significantly lower CHD risk in US men and women. The inverse associations may plateau at various intake levels for both total whole grain and individual whole grain foods. Findings from our study not only support the current dietary guideline that promotes total whole grain intake but also provide novel evidence that would further facilitate individuals’ choice of individual whole grains into their diet for CHD prevention.

## Supplementary Information

**Additional file 1: Table S1.** Pearson correlation coefficients between total whole grain and individual whole grain foods. **Table S2.** Pooled hazard ratios (95% confidence intervals) of coronary heart disease for individual whole grain food consumption in Nurses’ Health Study (1984-2016), Nurses’ Health Study II (1991-2017), and Health Professionals Follow-up Study (1986-2016). **Table S3.** Pooled hazard ratios (95% confidence intervals) of nonfatal myocardial infarction for individual whole grain food consumption in Nurses’ Health Study (1984-2016), Nurses’ Health Study II (1991-2017), and Health Professionals Follow-up Study (1986-2016). **Table S4.** Pooled hazard ratios (95% confidence intervals) of fatal coronary heart disease for individual whole grain food consumption in Nurses’ Health Study (1984-2016), Nurses’ Health Study II (1991-2017), and Health Professionals Follow-up Study (1986-2016). **Table S5.** Association between regular and light/fat free popcorn intake and risk of coronary heart disease in Nurses’ Health Study (2002-2016), Nurses’ Health Study II (2003-2017) and Health Professionals Follow-up study (2002-2016). **Table S6.** Pooled hazard ratios (95% confidence intervals) of coronary heart disease for individual whole grain food consumption in Nurses’ Health Study (1984-2016), Nurses’ Health Study II (1991-2017), Health Professionals Follow-up Study (1986-2016) using baseline intake, simple updated intake, or 4-year lag consumption. **Table S7.** Pooled hazard ratios (95% confidence intervals) of coronary heart disease for individual whole grain food consumption in Nurses’ Health Study (1984-2016), Nurses’ Health Study II (1991-2017), Health Professionals Follow-up Study (1986-2016) in analyses that resumed dietary update after 8 years of chronic diseases occurrence. **Figure S1.** Association between total whole grains and coronary heart disease risk stratified by body mass index, family history of diabetes, physical activity, and smoking status.

## Data Availability

The data underlying this article will be shared on reasonable request to the corresponding author.

## Abbreviations

AHEI: Alternative healthy eating index
BMI: Body mass index
CHD: Coronary heart disease
CI: Confidence interval
HPFS: Health Professionals Follow-up Study
HR: Hazard ratio
METs: Metabolic equivalent
MI: Myocardial infarction
NDI: National Death Index
NHS: Nurses’ Health Study
RCTs: Randomized clinical trials
sFFQ: Semi-quantitative food frequency questionnaire
