# Supplementary material for: Intake of whole grain foods and risk of coronary heart disease in US men and women
Source: BMC Med. 2022 Jun 10;20:192. doi: 10.1186/s12916-022-02396-z (PMC9185912; doi:10.1186/s12916-022-02396-z)
Supplement: Supplementary file 1 — Additional file 1: Table S1. Pearson correlation coefficients between total whole grain and individual whole grain foods. Table S2. Pooled hazard ratios (95% confidence intervals) of coronary heart disease for individual whole grain food consumption in Nurses’ Health Study (1984-2016), Nurses’ Health Study II (1991-2017), and Health Professionals Follow-up Study (1986-2016). Table S3. Pooled hazard ratios (95% confidence intervals) of nonfatal myocardial infarction for individual whole grain food consumption in Nurses’ Health Study (1984-2016), Nurses’ Health Study II (1991-2017), and Health Professionals Follow-up Study (1986-2016). Table S4. Pooled hazard ratios (95% confidence intervals) of fatal coronary heart disease for individual whole grain food consumption in Nurses’ Health Study (1984-2016), Nurses’ Health Study II (1991-2017), and Health Professionals Follow-up Study (1986-2016). Table S5. Association between regular and light/fat free popcorn intake and risk of coronary heart disease in Nurses’ Health Study (2002-2016), Nurses’ Health Study II (2003-2017) and Health Professionals Follow-up study (2002-2016). Table S6. Pooled hazard ratios (95% confidence intervals) of coronary heart disease for individual whole grain food consumption in Nurses’ Health Study (1984-2016), Nurses’ Health Study II (1991-2017), Health Professionals Follow-up Study (1986-2016) using baseline intake, simple updated intake, or 4-year lag consumption. Table S7. Pooled hazard ratios (95% confidence intervals) of coronary heart disease for individual whole grain food consumption in Nurses’ Health Study (1984-2016), Nurses’ Health Study II (1991-2017), Health Professionals Follow-up Study (1986-2016) in analyses that resumed dietary update after 8 years of chronic diseases occurrence. Figure S1. Association between total whole grains and coronary heart disease risk stratified by body mass index, family history of diabetes, physical activity, and smoking status. [file 12916_2022_2396_MOESM1_ESM.docx]

| Table S1. Pearson correlation coefficients between total whole grain and individual whole grain foods^*^ | | | | | | | | |
| --- | --- | --- | --- | --- | --- | --- | --- | --- |
|  | Total whole grain | Breakfast cereal | Dark bread | Oatmeal | Brown rice | Added bran | Wheat germ | Popcorn |
| Total whole grain | 1 | 0.55 | 0.29 | 0.44 | 0.47 | 0.21 | 0.17 | 0.22 |
| Breakfast cereal | - | 1 | 0.13 | 0.06 | 0.05 | 0.11 | 0.06 | 0.02 |
| Dark bread | - | - | 1 | 0.11 | 0.12 | 0.08 | 0.07 | 0.02 |
| Oatmeal | - | - | - | 1 | 0.14 | 0.11 | 0.11 | 0.02 |
| Brown rice | - | - | - | - | 1 | 0.09 | 0.10 | 0.06 |
| Added bran | - | - | - | - | - | 1 | 0.29 | 0.02 |
| Wheat germ | - | - | - | - | - | - | 1 | 0.01 |
| Popcorn | - | - | - | - | - | - | - | 1 |
| ^*^P<0.0001 for all Pearson correlation coefficients. Data from three cohorts were combined. | | | | | | | | |

| **Table S2.** Pooled hazard ratios (95% confidence intervals) of coronary heart disease for individual whole grain food consumption in Nurses’ Health Study (1984-2016), Nurses’ Health Study II (1991-2017), and Health Professionals Follow-up Study (1986-2016). | | | | | | |
| --- | --- | --- | --- | --- | --- | --- |
|  | Consumption levels | | | | P trend | Per one daily serving |
|  | < 1 serving/month | 1 serving/month -  1 serving/week | 1 serving/week –  4-6 servings/week | ≥1 servings/d |  |  |
| **Cold breakfast cereal** |  |  |  |  |  |  |
| NHS |  |  |  |  |  |  |
| Cases/person-time | 1,742/733,887 | 769/399,569 | 995/667,355 | 395/189,496 |  |  |
| Multivariable adjusted model * | 1.00 | 0.91(0.84,0.99) | 0.72(0.67,0.78) | 0.90(0.81,1.01) | <0.001 | 0.82(0.73,0.92) |
| NHSII |  |  |  |  |  |  |
| Cases/person-time | 315/617,605 | 195/495,012 | 233/912,482 | 74/306,965 |  |  |
| Multivariable adjusted model * | 1.00 | 0.84(0.70,1.00) | 0.63(0.53,0.75) | 0.63(0.49,0.82) | <.0001 | 0.56(0.43,0.73) |
| HPFS |  |  |  |  |  |  |
| Cases/person-time | 1,762/307,634 | 841/175,269 | 1,257/295,802 | 883/196,388 |  |  |
| Multivariable adjusted model* | 1.00 | 0.93(0.85,1.01) | 0.80(0.74,0.86) | 0.82(0.76,0.89) | 0.001 | 0.84(0.78,0.91) |
| P for heterogeneity | 0.02 | | | | | |
| **Dark bread** |  |  |  |  |  |  |
| NHS |  |  |  |  |  |  |
| Cases/person-time | 470/200,326 | 846/393,359 | 1,225/736,365 | 1,360/660,257 |  |  |
| Multivariable adjusted model * | 1.00 | 1.02(0.91,1.15) | 0.83(0.74,0.92) | 0.91(0.81,1.01) | 0.63 | 0.99(0.95,1.03) |
| NHSII |  |  |  |  |  |  |
| Cases/person-time | 122/276,456 | 208/478,771 | 286/906,256 | 201/670,580 |  |  |
| Multivariable adjusted model * | 1.00 | 1.09(0.87,1.36) | 0.89(0.72,1.11) | 0.87(0.69,1.10) | 0.10 | 0.91(0.81,1.02) |
| HPFS |  |  |  |  |  |  |
| Cases/person-time | 600/108,401 | 1,013/198,814 | 1,315/303,770 | 1,815/364,108 |  |  |
| Multivariable adjusted model * | 1.00 | 1.05(0.95,1.16) | 0.88(0.80,0.98) | 0.94(0.86,1.04) | 0.81 | 1.00(0.96,1.03) |
| P for heterogeneity | 0.31 | | | | | |
| **Popcorn** |  |  |  |  |  |  |
| NHS |  |  |  |  |  |  |
| Cases/person-time | 1,641/676,052 | 1,682/937,379 | 493/330,387 | 85/46,489 |  |  |
| Multivariable adjusted model * | 1.00 | 0.97(0.91,1.04) | 0.94(0.85,1.05) | 1.12(0.90,1.40) | 0.35 | 1.07(0.93,1.22) |
| NHSII |  |  |  |  |  |  |
| Cases/person-time | 104/313,525 | 448/1,259,692 | 192/638,973 | 73/119,874 |  |  |
| Multivariable adjusted model * | 1.00 | 1.13(0.91,1.40) | 0.91(0.71,1.16) | 1.51(1.11,2.06) | 0.19 | 1.12(0.95,1.32) |
| HPFS |  |  |  |  |  |  |
| Cases/person-time | 1,777/284,891 | 2,088/465,566 | 696/181,909 | 182/42,727 |  |  |
| Multivariable adjusted model * | 1.00 | 0.98(0.92,1.05) | 0.91(0.83,0.99) | 0.98(0.84,1.14) | 0.32 | 0.95(0.86,1.05) |
| P for heterogeneity | 0.18 | | | | | |
|  | < 1 serving/month | 1 serving/month -  1 serving/week | ≥2 servings/week |  |  |  |
| **Oatmeal** |  |  |  |  |  |  |
| NHS |  |  |  |  |  |  |
| Cases/person-time | 2,203/1,069,590 | 1,311/678,119 | 387/242,598 | - |  |  |
| Multivariable adjusted model * | 1.00 | 0.98(0.91,1.05) | 0.79(0.70,0.88) | - | 0.61 | 0.94(0.75,1.19) |
| NHSII |  |  |  |  |  |  |
| Cases/person-time | 415/1,009,274 | 320/949,289 | 82/373,501 | - |  |  |
| Multivariable adjusted model * | 1.00 | 0.90(0.78,1.05) | 0.62(0.49,0.80) | - | 0.008 | 0.44(0.23,0.81) |
| HPFS |  |  |  |  |  |  |
| Cases/person-time | 2,625/522,842 | 1,432/303,485 | 686/148,767 |  |  |  |
| Multivariable adjusted model * | 1.00 | 0.93(0.87,0.99) | 0.81(0.74,0.88) |  | 0.02 | 0.84(0.72,0.97) |
| P for heterogeneity | 0.07 | | | | | |
| **Brown rice** |  |  |  |  |  |  |
| NHS |  |  |  |  |  |  |
| Cases/person-time | 2,854/1,342,075 | 937/564,867 | 110/83,364 |  |  |  |
| Multivariable adjusted model * | 1.00 | 0.93(0.86,1.01) | 0.82(0.67,1.00) |  | 0.22 | 0.75(0.47,1.19) |
| NHSII |  |  |  |  |  |  |
| Cases/person-time | 494/1,210,105 | 269/922,677 | 54/199,281 |  |  |  |
| Multivariable adjusted model * | 1.00 | 0.85(0.73,0.99) | 0.83(0.62,1.11) |  | 0.38 | 0.72(0.34,1.51) |
| HPFS |  |  |  |  |  |  |
| Cases/person-time | 2,654/468,834 | 1,833/425,954 | 256/80,305 |  |  |  |
| Multivariable adjusted model * | 1.00 | 0.93(0.87,0.99) | 0.76(0.67,0.87) |  | 0.06 | 0.77(0.58,1.02) |
| P for heterogeneity | 0.98 | | | | | |
| **Added bran** |  |  |  |  |  |  |
| NHS |  |  |  |  |  |  |
| Cases/person-time | 3,128/1,517,452 | 419/250,212 | 354/222,643 |  |  |  |
| Multivariable adjusted model * | 1.00 | 0.90(0.81,1.00) | 0.82(0.73,0.92) |  | 0.74 | 0.98(0.86,1.11) |
| NHSII |  |  |  |  |  |  |
| Cases/person-time | 712/1,944,171 | 83/262,754 | 22/125,139 |  |  |  |
| Multivariable adjusted model * | 1.00 | 0.96(0.76,1.21) | 0.52(0.34,0.79) |  | 0.07 | 0.34(0.15,0.74) |
| HPFS |  |  |  |  |  |  |
| Cases/person-time | 3,616/722,494 | 584/131,772 | 543/120,828 |  |  |  |
| Multivariable adjusted model * | 1.00 | 0.94(0.86,1.02) | 0.86(0.79,0.95) |  | 0.007 | 0.89(0.82,0.97) |
| P for heterogeneity | 0.02 | | | | | |
| **Wheat germ** |  |  |  |  |  |  |
| NHS |  |  |  |  |  |  |
| Cases/person-time | 3,576/1,783,890 | 223/140,336 | 102/66,081 |  |  |  |
| Multivariable adjusted model * | 1.00 | 0.95(0.83,1.09) | 0.87(0.71,1.07) |  | 0.59 | 0.92(0.69,1.24) |
| NHSII |  |  |  |  |  |  |
| Cases/person-time | 780/2,157,074 | 32/139,601 | 5/35,389 |  |  |  |
| Multivariable adjusted model * | 1.00 | 0.80(0.56,1.15) | 0.48(0.20,1.17) |  | 0.21 | 0.32(0.06,1.88) |
| HPFS |  |  |  |  |  |  |
| Cases/person-time | 4,289/872,351 | 285/64,489 | 169/38,254 |  |  |  |
| Multivariable adjusted model * | 1.00 | 0.97(0.86,1.10) | 0.89(0.76,1.04) |  | 0.08 | 0.85(0.71,1.02) |
| P for heterogeneity | 0.49 | | | | | |
| *Adjusted for age (years), ethnicity (white, African American, Asian, others), body mass index (calculated as weight in kilograms divided by height in meters squared) (<21.0, 21.0-22.9, 23.0-24.9, 25.0-26.9, 27.0-29.9, 30.0-32.9, 33.0-34.9, or ≥35.0 kg/m^2^), smoking status (never smoked, past smoker, currently smoke 1-14 cigarettes per day, 15-24 cigarettes per day, or ≥25 cigarettes per day), alcohol intake (0, 0.1-4.9, 5.0-9.9, 10.0-14.9, 15.0-29.9, and ≥30.0 g/d), baseline diabetes, multivitamin use (yes, no), physical activity (quintiles), modified alternative healthy eating index (quintiles, whole grain component was excluded), total energy (quintiles), and family history of MI. For women, postmenopausal hormone use (never, former, or current hormone use, or missing), and oral contraceptive use were further adjusted. | | | | | | |

| Table S3. Pooled hazard ratios (95% confidence intervals) of nonfatal myocardial infarction for individual whole grain food consumption in Nurses’ Health Study (1984-2016), Nurses’ Health Study II (1991-2017), and Health Professionals Follow-up Study (1986-2016). | | | | | | |
| --- | --- | --- | --- | --- | --- | --- |
|  | Consumptions | | | | P trend | Per one daily serving |
|  | < 1 serving/month | 1 serving/month -  1 serving/week | 1 serving/week –  4-6 servings/week | ≥1 servings/d |  |  |
| Whole grain cold breakfast cereal |  |  |  |  |  |  |
| Cases/person-time | 2,332/1,659,139 | 1,159/1,069,855 | 1,612/1,875,645 | 775/692,851 |  |  |
| Multivariable adjusted model* | 1.00 | 0.91 (0.85, 0.98) | 0.77 (0.72, 0.83) | 0.83 (0.76, 0.90) | <.0001 | 0.82 (0.76, 0.89) |
| Dark bread |  |  |  |  |  |  |
| Cases/person-time | 727/585,187 | 1,287/1,070,950 | 1,814/1,946,399 | 2,050/1,694,954 |  |  |
| Multivariable adjusted model* | 1.00 | 1.02 (0.93, 1.12) | 0.85 (0.78, 0.93) | 0.91 (0.83, 0.99) | 0.76 | 1.01 (0.97, 1.04) |
| Popcorn |  |  |  |  |  |  |
| Cases/person-time | 1,957/1,274,479 | 2,757/2,662,647 | 951/1,151,275 | 213/209,091 |  |  |
| Multivariable adjusted model* | 1.00 | 1.01 (0.96, 1.08) | 0.95 (0.88, 1.03) | 1.06 (0.91, 1.24) | > 0.99 | 1.00 (0.91, 1.10) |
|  | < 1 serving/month | 1 serving/month -  1 serving/week | ≥2 servings/week |  |  |  |
| Oatmeal |  |  |  |  |  |  |
| Cases/person-time | 3,290/2,601,716 | 1,942/1,930,901 | 646/764,873 |  |  |  |
| Multivariable adjusted model* | 1.00 | 0.94 (0.88, 0.99) | 0.75 (0.68, 0.82) |  | 0.007 | 0.79 (0.66, 0.94) |
| Brown rice |  |  |  |  |  |  |
| Cases/person-time | 3,758/3,021,031 | 1,868/1,913,504 | 252/362,955 |  |  |  |
| Multivariable adjusted model* | 1.00 | 0.91 (0.85, 0.96) | 0.76 (0.66, 0.86) |  | 0.003 | 0.62 (0.45, 0.85) |
| Added bran |  |  |  |  |  |  |
| Cases/person-time | 4,679/4,184,134 | 658/644,743 | 541/468,613 |  |  |  |
| Multivariable adjusted model* | 1.00 | 0.91 (0.84, 0.99) | 0.86 (0.79, 0.94) |  | 0.02 | 0.90 (0.82, 0.99) |
| Wheat germ |  |  |  |  |  |  |
| Cases/person-time | 5,411/4,813,338 | 321/344,426 | 146/139,726 |  |  |  |
| Multivariable adjusted model* | 1.00 | 0.92 (0.82, 1.03) | 0.83 (0.70, 0.98) |  | 0.08 | 0.81 (0.65, 1.02) |
| *Adjusted for age (years), ethnicity (white, African American, Asian, others), body mass index (calculated as weight in kilograms divided by height in meters squared) (<21.0, 21.0-22.9, 23.0-24.9, 25.0-26.9, 27.0-29.9, 30.0-32.9, 33.0-34.9, or ≥35.0 kg/m^2^), smoking status (never smoked, past smoker, currently smoke 1-14 cigarettes per day, 15-24 cigarettes per day, or ≥25 cigarettes per day), alcohol intake (0, 0.1-4.9, 5.0-9.9, 10.0-14.9, 15.0-29.9, and ≥30.0 g/d), baseline diabetes, multivitamin use (yes, no), physical activity (quintiles), modified alternative healthy eating index (quintiles, whole grain component was excluded), total energy (quintiles), and family history of MI. For women, postmenopausal hormone use (never, former, or current hormone use, or missing), and oral contraceptive use were further adjusted.  Study estimates from three cohorts were pooled using a fixed effects model. | | | | | | |

| Table S4. Pooled hazard ratios (95% confidence intervals) of fatal coronary heart disease for individual whole grain food consumption in Nurses’ Health Study (1984-2016), Nurses’ Health Study II (1991-2017), and Health Professionals Follow-up Study (1986-2016). | | | | | | |
| --- | --- | --- | --- | --- | --- | --- |
|  | Consumptions | | | | P trend | Per one daily serving |
|  | < 1 serving/month | 1 serving/month -  1 serving/week | 1 serving/week –  4-6 servings/week | ≥1 servings/d |  |  |
| Whole grain cold breakfast cereal |  |  |  |  |  |  |
| Cases/person-time | 1,487/1,661,280 | 646/1,070,938 | 873/1,877,191 | 577/693,564 |  |  |
| Multivariable adjusted model^*^ | 1.00 | 0.91 (0.83, 1.00) | 0.71 (0.65, 0.78) | 0.84 (0.76, 0.93) | <.0001 | 0.80 (0.73, 0.88) |
| Dark bread |  |  |  |  |  |  |
| Cases/person-time | 465/585,859 | 780/1,072,170 | 1,012/1,948,063 | 1,326/1,696,881 |  |  |
| Multivariable adjusted model^*^ | 1.00 | 1.07 (0.95, 1.20) | 0.87 (0.78, 0.98) | 0.93 (0.84, 1.04) | 0.11 | 0.97 (0.93, 1.01) |
| Popcorn |  |  |  |  |  |  |
| Cases/person-time | 1,565/1,276,280 | 1,461/2,665,232 | 430/1,152,168 | 127/209,291 |  |  |
| Multivariable adjusted model^*^ | 1.00 | 0.94 (0.87, 1.01) | 0.86 (0.77, 0.96) | 1.15 (0.96, 1.39) | 0.47 | 1.04 (0.93, 1.17) |
|  | < 1 serving/month | 1 serving/month -  1 serving/week | ≥2 servings/week |  |  |  |
| Oatmeal |  |  |  |  |  |  |
| Cases/person-time | 1,953/2,604,785 | 1,121/1,932,694 | 509/765,493 |  |  |  |
| Multivariable adjusted model^*^ | 1.00 | 0.97 (0.90, 1.05) | 0.85 (0.77, 0.94) |  | 0.28 | 0.91 (0.76, 1.08) |
| Brown rice |  |  |  |  |  |  |
| Cases/person-time | 2,244/3,024,534 | 1,171/1,915,247 | 168/363,190 |  |  |  |
| Multivariable adjusted model^*^ | 1.00 | 0.95 (0.89, 1.03) | 0.84 (0.72, 0.99) |  | 0.96 | 1.01 (0.73, 1.38) |
| Added bran |  |  |  |  |  |  |
| Cases/person-time | 2,777/4,188,486 | 428/645,348 | 378/469,139 |  |  |  |
| Multivariable adjusted model^*^ | 1.00 | 0.95 (0.86, 1.05) | 0.81 (0.73, 0.91) |  | 0.15 | 0.93 (0.85, 1.03) |
| Wheat germ |  |  |  |  |  |  |
| Cases/person-time | 3,234/4,818,371 | 219/344,735 | 130/139,865 |  |  |  |
| Multivariable adjusted model^*^ | 1.00 | 1.02 (0.89, 1.17) | 0.94 (0.79, 1.13) |  | 0.40 | 0.91 (0.74, 1.13) |
| *Adjusted for age (years), ethnicity (white, African American, Asian, others), body mass index (calculated as weight in kilograms divided by height in meters squared) (<21.0, 21.0-22.9, 23.0-24.9, 25.0-26.9, 27.0-29.9, 30.0-32.9, 33.0-34.9, or ≥35.0 kg/m^2^), smoking status (never smoked, past smoker, currently smoke 1-14 cigarettes per day, 15-24 cigarettes per day, or ≥25 cigarettes per day), alcohol intake (0, 0.1-4.9, 5.0-9.9, 10.0-14.9, 15.0-29.9, and ≥30.0 g/d), baseline diabetes, multivitamin use (yes, no), physical activity (quintiles), modified alternative healthy eating index (quintiles, whole grain component was excluded), total energy (quintiles), and family history of MI. For women, postmenopausal hormone use (never, former, or current hormone use, or missing), and oral contraceptive use were further adjusted.  Study estimates from three cohorts were pooled using a fixed effects model. | | | | | | |

| Table S5. Association between regular and light/fat free popcorn intake and risk of coronary heart disease in Nurses’ Health Study (2002-2016), Nurses’ Health Study II (2003-2017) and Health Professionals Follow-up study (2002-2016). | | | | | | | | |
| --- | --- | --- | --- | --- | --- | --- | --- | --- |
|  | Regular popcorn | | | | Light, fat free popcorn | | | |
|  | <1 serving/mo | 1 serving/mo –  1 serving/wk | > 2 servings/wk | P trend | <1 serving/mo | 1 serving/mo –  1 serving/wk | > 2 servings/wk | P trend |
| NHS |  |  |  |  |  |  |  |  |
| Cases/person-years | 684/339,502 | 147/95,568 | 18/11,166 |  | 612/298,870 | 199/121,392 | 38/25,975 |  |
| Multivariable-adjusted model 1* | 1.00 | 0.90(0.75,1.08) | 0.96(0.59,1.54) | 0.64 | 1.00 | 1.01(0.85,1.19) | 1.05(0.75,1.47) | 0.77 |
| Multivariable-adjusted model 2* | 1.00 | 0.90(0.75,1.08) | 0.95(0.59,1.53) | 0.63 | 1.00 | 1.01(0.86,1.20) | 1.05(0.75,1.47) | 0.75 |
| NHSII |  |  |  |  |  |  |  |  |
| Cases/person-years | 178/463,937 | 133/324,026 | 26/45,725 |  | 208/415,108 | 100/336,327 | 29/82,253 |  |
| Multivariable-adjusted model 1* | 1.00 | 1.14(0.90,1.43) | 1.58(1.04,2.41) | 0.01 | 1.00 | 0.66(0.52,0.84) | 0.77(0.52,1.14) | 0.31 |
| Multivariable-adjusted model 2* | 1.00 | 1.15(0.92,1.45) | 1.56(1.02,2.37) | 0.01 | 1.00 | 0.66(0.52,0.84) | 0.75(0.51,1.12) | 0.26 |
| HPFS |  |  |  |  |  |  |  |  |
| Cases/person-years | 531/123,551 | 303/75,519 | 41/10,870 |  | 657/143,389 | 185/56,098 | 33/10,453 |  |
| Multivariable-adjusted model 1* | 1.00 | 1.07(0.92,1.23) | 0.98(0.71,1.35) | 0.10 | 1.00 | 0.87(0.74,1.03) | 0.88(0.62,1.25) | > 0.99 |
| Multivariable-adjusted model 2* | 1.00 | 1.08(0.93,1.25) | 0.98(0.71,1.35) | 0.08 | 1.00 | 0.86(0.73,1.02) | 0.89(0.62,1.27) | 0.57 |
| Pooled† |  |  |  |  |  |  |  |  |
| Multivariable-adjusted model 1* | 1.00 | 1.02 (0.90, 1.16) | 1.13 (0.83, 1.55) | 0.14 | 1.00 | 0.84 (0.68, 1.05) | 0.91 (0.74, 1.11) | 0.81 |
| Multivariable-adjusted model 2* | 1.00 | 1.03 (0.90, 1.18) | 1.12 (0.83, 1.52) | 0.12 | 1.00 | 0.85 (0.68, 1.05) | 0.90 (0.73, 1.11) | 0.49 |
| *Multivariable-adjusted model 1 adjusted for age, race (white, African-American, Asian, others), multivitamin use, family history of myocardial infarction, baseline diabetes, alcohol consumption (Abstainer, 0-4.9g/d, 5-9.9g/d, 10-14.9g/d, 15-29.9g/d and $\boldsymbol{\geq}$30g/d), modified alternative healthy eating index with whole grain excluded (quintiles), smoking status (never smoker, past smoker, current 1-14 cigarettes/d, current 15-24 cigarettes/d and$\boldsymbol{\geq}$25 cigarettes/d), body mass index (<21.0, 21.0-22.9, 23.0-24.9, 25.0-26.9, 27.0-29.9, 30.0-32.9, 33.0-34.9, or ≥35.0 kg/m^2^), oral contraceptive use (women only), hormone use (women only, pre-menopausal, post-menopausal with never use, current use and past use) and total energy (quintiles).  Multivariable-adjusted model 2 mutually adjusted for regular popcorn and light/fat free. | | | | | | | | |
| Study estimates from three cohorts were pooled using a fixed effects model | | | | | | | | |

| Table S6. Pooled hazard ratios (95% confidence intervals) of coronary heart disease for individual whole grain food consumption in Nurses’ Health Study (1984-2016), Nurses’ Health Study II (1991-2017), Health Professionals Follow-up Study (1986-2016) using baseline intake, simple updated intake, or 4-year lag consumption. | | | | | | |
| --- | --- | --- | --- | --- | --- | --- |
|  | Consumption levels | | | | P trend | Per one daily serving |
|  | < 1 serving/month | 1 serving/month -  1 serving/week | 1 serving/week –  4-6 servings/week | ≥1 servings/d |  |  |
| Whole grain cold breakfast cereal |  |  |  |  |  |  |
| Baseline intake |  |  |  |  |  |  |
| Cases/person-time | 4,486/2,229,183 | 1,841/1,166,893 | 1,594/986,265 | 1,540/915,122 |  |  |
| Multivariable adjusted model^*^ | 1.00 | 0.93 (0.88, 0.98) | 0.88 (0.83, 0.93) | 0.86 (0.81, 0.92) | <.0001 | 0.87 (0.82, 0.92) |
| Simple updated intake |  |  |  |  |  |  |
| Cases/person-time | 3,962/1,973,470 | 1,654/992,013 | 1,516/942,663 | 1,543/887,604 |  |  |
| Multivariable adjusted model^*^ | 1.00 | 0.98 (0.92, 1.04) | 0.86 (0.81, 0.91) | 0.83 (0.78, 0.88) | <.0001 | 0.82 (0.77, 0.87) |
| Lag 4 years |  |  |  |  |  |  |
| Cases/person-time | 3,071/1,380,134 | 1,487/889,691 | 2,061/1,545,194 | 1,122/583,307 |  |  |
| Multivariable adjusted model^*^ | 1.00 | 0.93 (0.87, 0.99) | 0.79 (0.75, 0.84) | 0.85 (0.80, 0.92) | <.0001 | 0.83 (0.77, 0.89) |
| Dark bread |  |  |  |  |  |  |
| Baseline intake |  |  |  |  |  |  |
| Cases/person-time | 1,456/832,919 | 2,462/1,385,668 | 2,131/1,271,305 | 3,412/1,807,573 |  |  |
| Multivariable adjusted model^*^ | 1.00 | 1.06 (0.99, 1.13) | 0.97 (0.90, 1.04) | 0.97 (0.91, 1.03) | 0.35 | 0.99 (0.97, 1.01) |
| Simple updated intake |  |  |  |  |  |  |
| Cases/person-time | 1,298/698,342 | 2,174/1,242,776 | 1,934/1,166,125 | 3,108/1,525,115 |  |  |
| Multivariable adjusted model^*^ | 1.00 | 1.05 (0.97, 1.12) | 0.95 (0.88, 1.02) | 0.98 (0.92, 1.05) | 0.41 | 0.99 (0.96, 1.01) |
| Lag 4 years |  |  |  |  |  |  |
| Cases/person-time | 974/490,062 | 1,673/896,811 | 2,340/1,604,093 | 2,754/1,407,361 |  |  |
| Multivariable adjusted model^*^ | 1.00 | 1.02 (0.94, 1.10) | 0.88 (0.81, 0.95) | 0.91 (0.84, 0.98) | 0.90 | 0.99 (0.96, 1.02) |
| Popcorn |  |  |  |  |  |  |
| Baseline intake |  |  |  |  |  |  |
| Cases/person-time | 3,988/1,568,119 | 4,224/2,770,757 | 872/663,486 | 377/295,103 |  |  |
| Multivariable adjusted model^*^ | 1.00 | 1.00 (0.95, 1.04) | 1.05 (0.97, 1.14) | 1.01 (0.91, 1.13) | 0.67 | 1.01 (0.95, 1.08) |
| Simple updated intake |  |  |  |  |  |  |
| Cases/person-time | 3,525/1,447,916 | 3,834/2,414,421 | 844/572,874 | 358/226,540 |  |  |
| Multivariable adjusted model^*^ | 1.00 | 0.99 (0.94, 1.04) | 1.03 (0.95, 1.11) | 1.05 (0.94, 1.18) | 0.36 | 1.03 (0.96, 1.11) |
| Lag 4 years |  |  |  |  |  |  |
| Cases/person-time | 2,803/1,040,037 | 3,475/2,225,202 | 1,174/956,493 | 289/176,596 |  |  |
| Multivariable adjusted model^*^ | 1.00 | 0.97 (0.92, 1.03) | 0.93 (0.87, 1.00) | 1.07 (0.94, 1.21) | 0.99 | 1.00 (0.92, 1.08) |
|  | < 1 serving/month | 1 serving/month -  1 serving/week | ≥2 servings/week |  |  |  |
| Oatmeal |  |  |  |  |  |  |
| Cases/person-time |  |  |  |  |  |  |
| Baseline intake |  |  |  |  |  |  |
| Cases/person-time | 5,818/3,060,083 | 2,904/1,845,979 | 739/391,402 |  |  |  |
| Multivariable adjusted model^*^ | 1.00 | 0.98 (0.93, 1.02) | 0.96 (0.89, 1.04) |  | 0.48 | 1.04 (0.93, 1.16) |
| Simple updated intake |  |  |  |  |  |  |
| Cases/person-time | 4,945/2,465,108 | 2,756/1,690,101 | 853/499,508 |  |  |  |
| Multivariable adjusted model^*^ | 1.00 | 0.95 (0.90, 0.99) | 0.79 (0.73, 0.86) |  | <.0001 | 0.73 (0.65, 0.82) |
| Lag 4 years |  |  |  |  |  |  |
| Cases/person-time | 4,268/2,171,657 | 2,518/1,608,909 | 955/617,759 |  |  |  |
| Multivariable adjusted model^*^ | 1.00 | 0.96 (0.91, 1.00) | 0.82 (0.77, 0.89) |  | 0.02 | 0.85 (0.74, 0.98) |
| Brown rice |  |  |  |  |  |  |
| Baseline intake |  |  |  |  |  |  |
| Cases/person-time | 6,374/3,354,525 | 2,792/1,721,139 | 295/221,801 |  |  |  |
| Multivariable adjusted model^*^ | 1.00 | 0.95 (0.91, 0.99) | 0.89 (0.79, 1.00) |  | 0.11 | 0.84 (0.68, 1.04) |
| Simple updated intake |  |  |  |  |  |  |
| Cases/person-time | 5,637/2,834,497 | 2,619/1,612,575 | 269/200,906 |  |  |  |
| Multivariable adjusted model^*^ | 1.00 | 0.93 (0.89, 0.98) | 0.83 (0.73, 0.95) |  | 0.004 | 0.71 (0.56, 0.89) |
| Lag 4 years |  |  |  |  |  |  |
| Cases/person-time | 4,850/2,510,599 | 2,527/1,589,505 | 364/298,225 |  |  |  |
| Multivariable adjusted model^*^ | 1.00 | 0.94 (0.89, 0.99) | 0.84 (0.75, 0.94) |  | 0.16 | 0.84 (0.66, 1.07) |
| Added bran |  |  |  |  |  |  |
| Baseline intake |  |  |  |  |  |  |
| Cases/person-time | 7,794/4,379,333 | 941/560,923 | 726/357,207 |  |  |  |
| Multivariable adjusted model^*^ | 1.00 | 0.93 (0.86, 0.99) | 0.90 (0.83, 0.97) |  | 0.01 | 0.92 (0.87, 0.98) |
| Simple updated intake |  |  |  |  |  |  |
| Cases/person-time | 6,785/3,930,700 | 835/423,608 | 599/270,303 |  |  |  |
| Multivariable adjusted model^*^ | 1.00 | 1.00 (0.92, 1.07) | 0.93 (0.85, 1.01) |  | 0.02 | 0.92 (0.86, 0.99) |
| Lag 4 years |  |  |  |  |  |  |
| Cases/person-time | 6,087/3,478,483 | 896/535,168 | 758/384,677 |  |  |  |
| Multivariable adjusted model^*^ | 1.00 | 0.94 (0.88, 1.01) | 0.86 (0.80, 0.94) |  | 0.02 | 0.91 (0.85, 0.98) |
| Wheat germ |  |  |  |  |  |  |
| Baseline intake |  |  |  |  |  |  |
| Cases/person-time | 8,724/4,860,583 | 520/335,196 | 217/101,684 |  |  |  |
|  | 1.00 | 0.97 (0.89, 1.06) | 0.88 (0.77, 1.01) |  | 0.08 | 0.88 (0.77, 1.02) |
| Simple updated intake |  |  |  |  |  |  |
| Cases/person-time | 7,814/4,316,541 | 448/250,991 | 181/78,511 |  |  |  |
| Multivariable adjusted model^*^ | 1.00 | 1.06 (0.96, 1.17) | 0.93 (0.80, 1.09) |  | 0.43 | 0.94 (0.80, 1.10) |
| Lag 4 years |  |  |  |  |  |  |
| Cases/person-time | 7,048/3,995,507 | 465/287,540 | 228/115,281 |  |  |  |
| Multivariable adjusted model^*^ | 1.00 | 0.99 (0.90, 1.09) | 0.88 (0.77, 1.01) |  | 0.18 | 0.89 (0.75, 1.06) |
| * Adjusted for age (years), ethnicity (white, African American, Asian, others), updated body mass index (calculated as weight in kilograms divided by height in meters squared) (<21.0, 21.0-22.9, 23.0-24.9, 25.0-26.9, 27.0-29.9, 30.0-32.9, 33.0-34.9, or ≥35.0 kg/m^2^), smoking status (never smoked, past smoker, currently smoke 1-14 cigarettes per day, 15-24 cigarettes per day, or ≥25 cigarettes per day), alcohol intake (0, 0.1-4.9, 5.0-9.9, 10.0-14.9, 15.0-29.9, and ≥30.0 g/d), baseline diabetes, multivitamin use (yes, no), physical activity (quintiles), modified alternative healthy eating index (quintiles, whole grain component was excluded), total energy (quintiles), and family history of MI. For women, postmenopausal hormone use (never, former, or current hormone use, or missing), and oral contraceptive use were further adjusted.  Study estimates from three cohorts were pooled using a fixed effects model. | | | | | | |

| Table S7. Pooled hazard ratios (95% confidence intervals) of coronary heart disease for individual whole grain food consumption in Nurses’ Health Study (1984-2016), Nurses’ Health Study II (1991-2017), Health Professionals Follow-up Study (1986-2016) in analyses that resumed dietary update after 8 years of chronic diseases occurrence. | | | | | | |
| --- | --- | --- | --- | --- | --- | --- |
|  | Consumption levels | | | | P trend | Per one daily serving |
|  | < 1 serving/month | 1 serving/month -  1 serving/week | 1 serving/week –  4-6 servings/week | ≥1 servings/d |  |  |
| Whole grain cold breakfast cereal |  |  |  |  |  |  |
| Cases/person-time | 3,172/1,456,562 | 1,702/1,031,078 | 3,428/2,185,526 | 1,159/624,298 |  |  |
| Multivariable adjusted model^*^ | 1.00 | 0.91 (0.86, 0.97) | 0.80 (0.76, 0.84) | 0.82 (0.77, 0.88) | <.0001 | 0.81 (0.75, 0.86) |
| Dark bread |  |  |  |  |  |  |
| Cases/person-time | 962/509,269 | 1,673/945,617 | 3,463/2,169,314 | 3,363/1,673,263 |  |  |
| Multivariable adjusted model^*^ | 1.00 | 1.03 (0.95, 1.12) | 0.90 (0.83, 0.97) | 0.94 (0.87, 1.01) | 0.60 | 0.99 (0.97, 1.02) |
| Popcorn |  |  |  |  |  |  |
| Cases/person-time | 3,224/1,211,879 | 4,077/2,577,603 | 1,872/1,325,424 | 288/182,560 |  |  |
| Multivariable adjusted model^*^ | 1.00 | 1.00 (0.95, 1.05) | 0.97 (0.91, 1.03) | 1.08 (0.95, 1.23) | 0.84 | 1.01 (0.93, 1.09) |
|  | < 1 serving/month | 1 serving/month -  1 serving/week | ≥2 servings/week |  |  |  |
| Oatmeal |  |  |  |  |  |  |
| Cases/person-time | 4,582/2,404,042 | 3,077/1,922,947 | 1,802/970,474 |  |  |  |
| Multivariable adjusted model^*^ | 1.00 | 0.97 (0.93, 1.02) | 0.87 (0.82, 0.92) |  | <0.001 | 0.84 (0.74, 0.95) |
| Brown rice |  |  |  |  |  |  |
| Cases/person-time | 5,750/2,935,633 | 3,153/1,948,543 | 558/413,287 |  |  |  |
| Multivariable adjusted model^*^ | 1.00 | 0.95 (0.90, 0.99) | 0.86 (0.79, 0.94) |  | 0.08 | 0.81 (0.64, 1.02) |
| Added bran |  |  |  |  |  |  |
| Cases/person-time | 7,170/4,122,070 | 1,136/657,451 | 1,155/517,944 |  |  |  |
| Multivariable adjusted model^*^ | 1.00 | 0.93 (0.87, 0.99) | 0.90 (0.84, 0.96) |  | 0.06 | 0.93 (0.87, 1.00) |
| Wheat germ |  |  |  |  |  |  |
| Resuming to update diet 8 years after occurrence of diseases |  |  |  |  |  |  |
| Cases/person-time | 8,609/4,810,868 | 514/334,360 | 338/152,236 |  |  |  |
| Multivariable adjusted model^*^ | 1.00 | 0.94 (0.86, 1.03) | 0.95 (0.85, 1.06) |  | 0.19 | 0.90 (0.76, 1.06) |
| * Adjusted for age (years), ethnicity (white, African American, Asian, others), updated body mass index (calculated as weight in kilograms divided by height in meters squared) (<21.0, 21.0-22.9, 23.0-24.9, 25.0-26.9, 27.0-29.9, 30.0-32.9, 33.0-34.9, or ≥35.0 kg/m^2^), smoking status (never smoked, past smoker, currently smoke 1-14 cigarettes per day, 15-24 cigarettes per day, or ≥25 cigarettes per day), alcohol intake (0, 0.1-4.9, 5.0-9.9, 10.0-14.9, 15.0-29.9, and ≥30.0 g/d), baseline diabetes, multivitamin use (yes, no), physical activity (quintiles), modified alternative healthy eating index (quintiles, whole grain component was excluded), total energy (quintiles), and family history of MI. For women, postmenopausal hormone use (never, former, or current hormone use, or missing), and oral contraceptive use were further adjusted.  Study estimates from three cohorts were pooled using a fixed effects model. | | | | | | |

**Figure S1.** Association between total whole grains and coronary heart disease risk stratified by body mass index, family history of diabetes, physical activity, and smoking status.^*†^


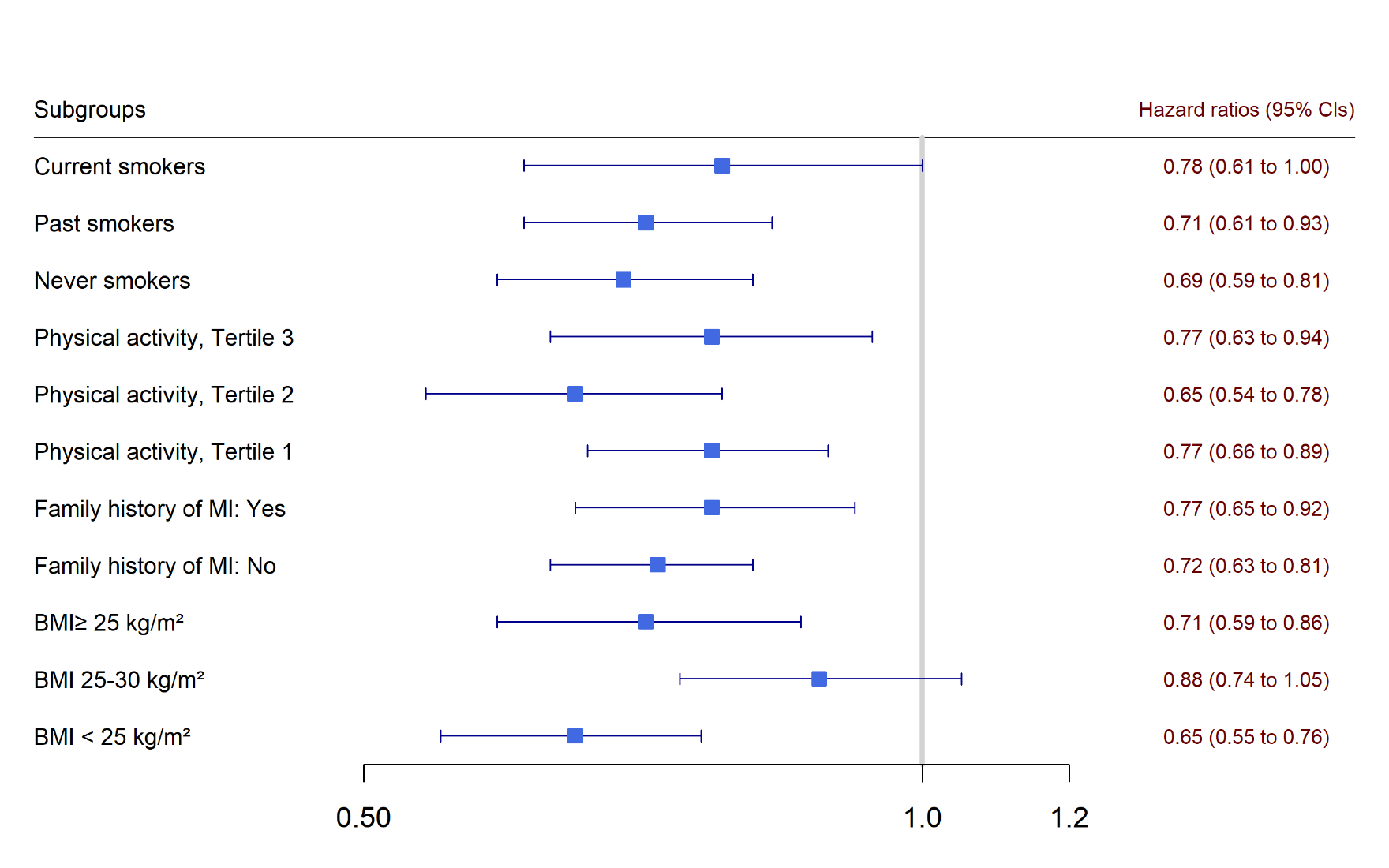


^*^Comparing extreme quintiles.

^†^Adjusted for same covariates as in Table 3 except for the stratification variable. Continuous BMI and physical activity were adjusted to minimize residual confounding.

Axis is in log scale.
